# Exploring Volatile Organic Compound Exposure and Its Association with Wheezing in Children under 36 Months: A Cross-Sectional Study in South Lisbon, Portugal

**DOI:** 10.3390/ijerph17186929

**Published:** 2020-09-22

**Authors:** Raquel Rodrigues dos Santos, João Gregório, Liliana Castanheira, Ana S. Fernandes

**Affiliations:** 1 CBIOS—Research Center for Biosciences and Health Technologies, Universidade Lusófona de Humanidades e Tecnologias, Campo Grande, 376, 1749-024 Lisboa, Portugal; raquel.santos@arslvt.min-saude.pt (R.R.d.S.); lilianacastanheira2@gmail.com (L.C.); ana.fernandes@ulusofona.pt (A.S.F.); 2 Administração Regional de Saúde de Lisboa e Vale do Tejo, I.P. Agrupamento de Centros de Saúde do Arco Ribeirinho, Unidade Saúde de Saúde Pública Arnaldo Sampaio, 2835-423 Lavradio, Portugal; 3 Administração Regional de Saúde de Lisboa e Vale do Tejo, I.P., Unidade Saúde Familiar do Dafundo, 1495-713 Dafundo, Portugal

**Keywords:** infants, wheezing, volatile organic compounds, bedrooms, parental smoking

## Abstract

Air quality and other environmental factors are gaining importance in public health policies. Some volatile organic compounds (VOCs) have been associated with asthma and symptoms of respiratory disease such as wheezing. The aim of this study was to measure the concentration of Total VOCs and assess their possible association with the occurrence of wheezing episodes in children under 36 months of age, in a region south of Lisbon, Portugal. A cross-sectional study was performed from October 2015 to March 2016. The sample of children under 36 months of age was selected by convenience, by inviting parents to take part in the study. A survey was applied to collect information on bedroom features, as well as to verify the occurrence of wheezing episodes. The indoor air quality parameters of bedrooms were measured using three 3M Quest^®^ EVM-7 environmental monitors. In total, 34.4% of infants had had wheezing episodes since birth, with 86.7% of these presenting at least one episode in the previous 12 months. Total VOC levels were above the reference values in 48% of the analyzed bedrooms. No significant association of VOC exposure in a domestic setting with episodes of wheezing was found. However, children living in households with smokers were 4 times more likely to develop wheezing episodes. Thus, this study provides relevant information that warrants further studies to assess infant exposure to indoor air pollution and parental smoking in a residential context.

## 1. Introduction

Reducing the burden of non-communicable and chronic respiratory diseases has become essential to advanced economies and for the sustainable development of many countries [1,2,3]. As chronic respiratory diseases such as asthma and other allergic disorders increase their prevalence [3,4], it has become vital to investigate the link between environmental effects and air quality and the respiratory health of a population, as highlighted in the intersection of the United Nations Sustainable Development Goals 3 and 11 [1,5]. Asthma and other chronic respiratory diseases usually have an early life onset [6]. The respiratory physiology of infants increases their vulnerability to air pollutants since they breathe more air per body weight than adults do, and their respiratory system is under development [7]. Wheezing, as a symptom of respiratory disease, is often associated with asthma [3]. It is a usual reason to seek a medical appointment, and it may appear on a recurring basis. Its prevalence has been increasing, particularly in developed countries [8], reinforcing its great relevance and impact on families and society due to the inherent costs [9,10]. The factors that influence the occurrence of wheezing/asthma in children are still a matter of debate. Whereas the increasing prevalence in developed countries has been attributed to urbanization/westernized lifestyle, obesity, and pollution [8], other authors defend the “hygiene hypothesis” suggesting that decreased exposure to unhygienic environments in early life may lead to increased prevalence of respiratory conditions [11]. In fact, studies in Amish and Hutterite children, as well as in European farm children, have demonstrated that early life exposure to the farm environment protects against asthma and allergy [12]. In Portugal, the global asthma prevalence is 6.8% [13], while a prevalence of 9.56% was found for the 0 to 7 year age group [14]. Childhood asthma in Portugal costs over €150 million per year (0.9% of the healthcare expenditure), corresponding to more than €900 per child [15].

Some volatile organic compounds (VOCs) have been associated with asthma and symptoms of respiratory disease [9]. VOCs are particularly relevant indoor air pollutants that come from several domestic sources including paints, floor and wall coverings, furniture, fabrics, mattresses, cleaning agents, air fresheners, or cosmetics [2,16]. As parents frequently remodel their babies’ bedrooms, the level of VOCs can increase substantially, impacting indoor air quality (IAQ) [7]. Moreover, young children spend most of their time at home, increasing the exposure to indoor air pollutants that may have considerable undesirable effects on their health [17,18]. Therefore, it is important to understand how exposure to VOCs in early life may play a role in the development of respiratory disease. Despite the higher susceptibility of infants and young children to respiratory diseases, research conducted on residential indoor air environments for this age group is scarce [7]. In a recent systematic review, Nurmatov et al. analyzed the possible association between increased residential VOC exposure and respiratory symptoms [2]. Considering the contradictory reports published in the literature and the insufficient quality of many available studies, it is not possible to draw a definite assessment on the implication of VOC exposure for the risk of developing and/or exacerbating asthma and allergy [2]. Thus, more work is required to clarify this relationship. Regarding the Portuguese context, although previous studies investigated IAQ in schools [19,20], information focused on early ages in a residential context is still missing.

To address this gap, the present study aimed to estimate the prevalence of wheezing in infants and its possible association with VOC exposure, by relating the levels of VOCs and other IAQ parameters in babies’ bedrooms with the number of wheezing episodes. Moreover, this evaluation would enable us to determine whether VOCs are within the reference values (see [21,22,23,24]); and finally, whether there is any correlation between the VOC levels and bedrooms’ or household characteristics.

## 2. Materials and Methods

To address the proposed aim, a cross-sectional study was conducted between October 2015 and March 2016. Two data collection tools were used: 3M Quest^®^ EVM-7 (3M Detection Solutions, Oconomowoc, WI, USA) environmental monitors and a newly developed survey [25]. Approval from Lusófona University and Tagus Valley Health Administration Ethics committees was obtained prior to any research activities (License no. 4/2015 and 11386/CES/2015 respectively). In the following sections, details are provided regarding study’s design.

### 2.1. Study Setting

**Figure 1:**
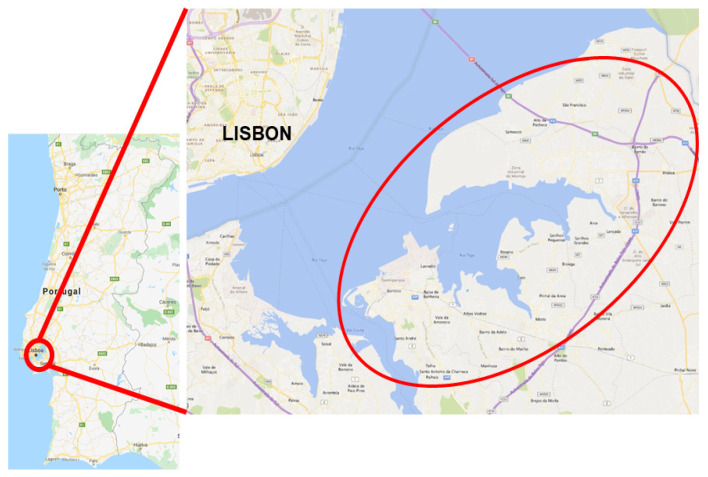
Map of the region where the study took place.

Study population were children aged 0–36 months, born in the Arco Ribeirinho region, in the south bank of river Tagus, Lisbon, Portugal (Figure 1). The choice of this age group was due to the lack of studies and also because this population is more likely to spend more time in the bedroom. The choice of this location was based on convenience and feasibility, since the main author was working in the region’s Public Health Unit. A Public Health Unit is a functional unit of a Healthcare Centers Clusters devoted to all matters regarding public health, epidemiological surveillance, health promotion, and disease prevention. These clusters are a major feature of the Portuguese Primary Health Care network, which include Family Health Units (FHUs) [26]. In these FHUs, several health care services are provided, including child health, maternal health, and pre-labor consultations. This cluster assists four municipalities with a total population of approximately 215,410 people. A total of 6151 babies were born in this region from 2012 through 2014.

### 2.2. Sampling Strategy

Due to the sensitive nature of the study, a choice was made to recruit a convenience sample at the child health consultations of the cluster’s 13 FHUs. A total of 57 health professionals (maternal health nurses and doctors) were trained for the recruiting procedure. All families with children under 36 months of age attending these consultations were invited to take part of the study. Parents were presented with a recruitment guide that provided answers to common doubts about VOCs and about study’s procedures. After assessing parents’ willingness to participate, an informed consent form was signed, and telephone contacts were registered in a separated form. The recruitment process started in July 2015 through September 2015, yielding a total of 269 subjects.

### 2.3. Data Collection

A survey was designed by performing a literature review of relevant studies in the PubMed database. Questions were aligned with the International Study of Asthma and Allergies in Childhood (ISAAC) [27]. In the end, 16 papers were used to support the survey’s design, as reported elsewhere [25]. The resulting survey was pre-tested by Environmental Health Officers in five families with babies in June 2015. Changes were done, and a decision to repeat the pre-test with the new version was made. After the final pre-test, some minor changes were made, resulting in the final version to be applied [25].

Data collection started in October 2015 lasting until March 2016. The choice of this period was made since it is the most likely to present higher indoor concentrations of VOCs [28,29]. Families were contacted by eight Environmental Health Officers who received specific training on the data collection tools, equipment, and communication with parents about respiratory physiology of infants. These professionals’ role was to perform measurements of IAQ parameters: total volatile organic compounds (TVOCs; mg/m^3^), carbon dioxide (CO_2_; ppm), carbon monoxide (CO; mg/m^3^), temperature (T; °C), and relative humidity (RH; %). These measurements were carried out using three 3M Quest^®^ EVM-7 environmental monitors. This equipment uses a photoionization detector to measure TVOCs, a non-dispersive infrared sensor for carbon dioxide, an electrochemical sensor to detect CO, a junction diode as a temperature sensor, and a capacitive humidity sensor. The chosen monitor was the one available at the Public Health Units, being one of the monitors certified by the Portuguese Institute of Quality (IPAC). Prior to the data collection, the instruments were calibrated for all the parameters under study by a company certified by IPAC.

The monitoring of air quality parameters was performed in the babies’ usual sleeping bedroom. It was done by positioning the monitor 1.5 m above the floor, near the center of the room, for at least 15 min, according to the Portuguese Technical Norm (NT) of the National System for Energy Certification of Buildings (SCE) NT-SCE-02, supported by International Organization for Standardization (ISO) ISO 16000-5:2007 and ISO 7730:2005. No one was allowed to be in the room during the monitoring, and all the doors and windows were kept closed. The same measurements were performed outside the house. While the monitoring occurred, the survey was administered. Finally, parents were verbally informed about the results of the monitoring.

### 2.4. Variables of Interest

Alongside with the IAQ measurements and bedroom characteristics collected in the survey, other variables of interest included sociodemographic variables such as the age of infants in months, sex, urbanization (“rural/urban”), house typology (“house/apartment”), and house occupation (number of people living in the household). Parents’ smoking habits and proximity to a pollution site (if a pollution site such as a highway, gas station, a factory, or a cow barn was visible or the smell could be noticed from inside or near the entrance of the house) were also registered in a categorical answer (“yes/no”). The main outcome of interest was the number of wheezing episodes in the last 12 months. A wheezing episode was defined as a period of continued audible wheezing. To account for more than one episode, episodes would have to be separated by at least one week without audible wheezing. Three categories (never/between one and three episodes/more than three episodes) were defined for the outcome variable “episodes of wheezing”. TVOC was redefined as a dummy variable, with a cutoff at the reference value (0.3 mg/m^3^) [23,24].

### 2.5. Statistical Analysis

Descriptive statistics and Shapiro–Wilk test to assess sample normality were used across variables. t-Student and Mann–Whitney tests to assess significance were conducted on continuous variables such as IAQ. Indoor/outdoor ratios were calculated for air quality parameters, by dividing the indoor value by the outdoor value—when both values were zero, the ratio was considered to be 1. The ratio was considered missing if the outside values were zero, and thus not included in the statistical analysis. Chi-square test with the Bonferroni correction was used to assess associations between categorical variables. Binary and multinomial logistic regressions were performed to assess the association of explanatory variables with the presence of wheezing. The significance level was set to *p* < 0.05.

All statistical analyses were performed in IBM software Statistical Package for Social Sciences (SPSS) version 23 (IBM Corp., Armonk, NY, USA).

## 3. Results

The 269 families available to participate were contacted by telephone to schedule the IAQ assessment and survey. During this telephone contact, we found that 12 families listed an unavailable contact, 44 stated that they wanted to quit, and 82 never answered the telephone. As a result, the survey and IAQ assessment were performed on a sample of 131 families (response rate: 48.7%).

Among the participant children, 68 (51.9%) were boys and 85 (64.9%) were infants under 1 year old. The mean age of the sample was 11.60 months. Although the boys were younger than the girls, the difference was not significant (*p* = 0.776). A total of 45 infants (34.4%) had had wheezing episodes, with 39 (86.7%) of these infants presenting at least one episode in the past 12 months. Wheezing was more prevalent in girls than in boys (Table 1), considering either episodes from birth or in the past 12 months. However, these differences were not significant (*p* = 0.108 and *p* = 0.267, respectively). The number of people living in the same house varied from 2 through 10, with most families (91.6%) having between 3 and 5 people. Most of our sample lived in an urban area apartment (80.1%), with the child’s bedroom located at least on the first floor or higher (Table 2).

Only 24.6% of families lived near a pollution site. In 30.5% of the households, there was at least one tobacco smoker, and in 29.8% of children’s bedrooms mold was visible. In spite of girls having bigger bedrooms, the differences were not significant.

In most bedrooms (68.7%), families opened the bedroom window at least once a day. More than half of the bedrooms were used to perform personal hygiene. One third of the bedrooms had recently washed clothes inside in the last 48 h. More families chose an oil radiator for heating purposes, although the big majority (85%) reported using no heating equipment at all. Only 4.6% reported having used an anti-moth product inside the bedroom in the 48 h prior to the IAQ measurements. Table 3 provides an overview of the IAQ parameters registered in our study and the IAQ parameters’ indoor/outdoor (I/O) ratios.

A large number of rooms had IAQ parameters outside the reference values, especially regarding temperature and relative humidity. All the parameters had an I/O ratio median of at least one, with means showing that, for the bedrooms with valid values, TVOC was double than that found outside while the indoor CO_2_ concentration was 5 times that found outside. After assumption tests, multiple logistic regressions were performed. Concerning house characteristics, only room area showed a significant association with the occurrence of wheezing episodes: wheezing episodes happened preferentially in smaller rooms, as we recently reported [30]. Moreover, lower temperatures (OR 0.679 [CI 95%: 0.517–0.892]; (*p* = 0.005)) and higher relative humidity (OR 1.093 [CI 95%: 1.007–1.185]; (*p* = 0.032)) were also associated with having 1 to 3 wheezing episodes.

Several variables were tested to assess the association with indoor TVOC concentrations, namely presence of in-house smokers, proximity to a pollution site, use of the bedroom to keep recently washed clothes, and the use of anti-moth products. Only the presence of smokers showed a significant association with TVOC concentration. Rooms in houses with smokers were associated with a higher likelihood of having TVOC concentrations above 0.3 mg/m^3^, adjusting for temperature, relative humidity, CO_2_, CO, and room size (aOR 3.251 [CI 95%: 1.333–7.933]; (*p* = 0.010)). Moreover, an association between the presence of indoor smokers and occurrence of wheezing episodes was found (χ^2^ = 12.322; *p* = 0.002). As for the presence of wheezing episodes and exposure to TVOCs, Table 4 shows that there is only a weak correlation. In the final adjusted models, the wheezing episodes showed a tendency to be related with TVOC above 0.3 mg/m^3^ but no significant association was found (Table 5). However, the regression model also showed that a baby living in a household with smokers is more likely to develop 1 to 3 wheezing episodes than one living in non-smokers’ houses [(aOR 4.202 [CI 95%: 1.122–15.625]; (*p* = 0.033)).

## 4. Discussion

This study aimed at assessing the association between TVOC exposure and episodes of wheezing in a sample of children under 36 months of age. To the best of our knowledge, this was an innovative study for Portugal, providing data from household IAQ measurements and the evaluation of its impact on respiratory disease incidence in a very specific population (children under 36 months).

The prevalence of wheezing was similar to that found in studies of this population elsewhere [31]. As the results illustrate, wheezing episodes were less prevalent in bedrooms with low levels of TVOCs. This result was expected, as the association between TVOCs and respiratory disease is already described in the literature [9,32]. Nevertheless, no significant association between levels of TVOCs and wheezing was found, possibly due to limitations that we discuss further ahead. Still, a significant association of low temperatures, high relative humidity, and presence of smokers with wheezing episodes was found. These findings add to the growing body of evidence about the synergistic effects of parental cigarette smoking and diverse air quality parameters on the occurrence of respiratory diseases and allergies [33,34,35]. Considering that our results show a significant association of smokers’ presence with higher concentrations of TVOCs, one can speculate that the reason for not finding a significant association between absence of wheezing and low levels of TVOCs was mainly due to the low number of participants and the insufficient air monitoring time. In spite of the methodology used in this study not allowing for the measurement of individual VOC, our findings compare with previous reports, confirming the importance of avoiding smoking habits to prevent the occurrence of respiratory disease in children under 36 months of age. A previous study performed in Portugal also found an association of wheezing episodes with toluene [19], a known constituent of cigarette smoke. Therefore, our results reinforce the importance of avoiding indoor smoking for parents with young children. Public health authorities’ role is now to tailor the message for parents to avoid such behavior.

Finally, the indoor/outdoor ratio of IAQ parameters found in this study should be better investigated in future studies. The relationship between indoor and outdoor parameters of air quality has been a matter of investigation, providing relevant information to understand the effect of outdoor sources on indoor exposure. Indeed, outdoor air contribution to indoor air quality seems to be related to the geographical location and respective weather. Some previous studies found indoor concentrations were much higher than outdoor concentrations for most VOCs [36]. Nevertheless, indoor exposure to VOCs may be more dependent on outdoor pollutants in temperate and warm latitudes, where building construction is generally less tight and doors and windows are opened more frequently, allowing for air exchange [37,38]. In fact, our data show that approximately 70% of the participants opened the bedroom window once or twice a day. The I/O ratios found also point to an insufficient capacity of these buildings to provide comfort. Our analysis did not provide details of building construction characteristics, but obtaining these data would help to assess the relation between thermic comfort and wheezing.

The innovative approach taken with this study, assessing infants’ exposure to TVOCs inside their bedroom, highlights the already reported importance of assessing air quality in the household [39,40]. For the Portuguese context, our findings add to the growing evidence on the necessity to regularly assess air quality both at home and occupational settings such as nurseries and schools [17,19,29,41]. However, this study has several limitations that must be addressed. Firstly, the choice of wheezing as the outcome variable could be questioned. Other symptoms such as cough or nasal allergy could be chosen [19]. The contradictory reports of the impact of TVOCs on respiratory disease found in the literature are partially due to the lack of standardization in assessing asthma-/allergy-related health outcomes, as identified by Nurmatov et al. [2]. In our study, wheezing was chosen as an outcome variable, since it is a very specific symptom of respiratory disease easily identifiable even by inexperienced parents. Another important point is that wheezing may be caused by different factors, other than exposure to VOCs. For example, since low temperatures and high values of relative humidity were found in our study, the impact of dust mite allergens might be especially relevant [42]. Parents’ allergy history or the presence of dust mite allergens may influence the occurrence of wheezing episodes and should be contemplated as explanatory variables in future investigations.

Secondly, parents may have over-reported wheezing episodes. The region of Arco Ribeirinho has a history of air pollution due to the presence of heavy industry, making this population highly aware of air pollution problems [41,43]. Nevertheless, it is important to highlight that no association was found in this sample between proximity to a pollution site and TVOC concentrations inside the bedroom. But since people are over alert, parents may have over-reported wheezing episodes when asked to recall the number of episodes in the past 12 months. One strategy to overcome this hurdle would be to recruit families when they visit the urgent health care public service due to an active wheezing episode. However, as parents may opt for a private health care service, our strategy seemed to be the most efficient in obtaining a large number of participants.

Furthermore, air sampling duration is also a limitation that has to be considered when interpreting our results. We recognize that the optimal sampling duration in order to assess exposure should be longer. Longer periods of sampling are usually used in most studies, but these assessments are seldom made in a residential context. The complexity of assessing household indoor VOCs is recognized and researchers’ usual choice to avoid such complexity is to develop tools such as surveys that identify sources of household VOCs [44]. In order to have some direct data, there was a need to compromise between the available monitoring time researchers had and the intrusion in a personal space. However, this weakened our results. Strategies to allow for longer monitoring times should be developed, taking into consideration that the environmental monitors are public devices that must be used under supervision.

Finally, although the sampling strategy yielded a convenient number of participants, in line or even above other studies carried out in residential context [7,36,38], a better compliance of the recruited families with study prosecution would benefit the results. In fact, the low number of participants may have contributed to the overrepresentation of parents who are interested in the issue and more likely to have children with respiratory diseases. Children of families who could not be re-contacted or refused participation were similar to participating children with respect to geographical area and age range, but no information on the occurrence of wheezing was available on non-respondents, since this information was collected during the visit of the Environmental Health Officers to the families’ homes. Thus, the representativeness of participants versus non-participants is uncertain. A better strategy to recall parents, less time between recruiting and study start or recruiting in other health services, may have contributed to a higher number of participants allowing for more significant results. Moreover, a random sampling strategy, a more sensitive meter, and registering buildings’ construction characteristics are suggested for future studies. In this way, extrapolation for the Arco Ribeirinho region would be possible.

## 5. Conclusions

This study reports on findings relative to a study on the association of total volatile organic compound concentrations in bedrooms of children under 36 months of age with wheezing in a region south of Lisbon, Portugal. As reported, a large number of rooms showed elevated concentrations of TVOCs and a correlation with wheezing episodes, even if not significant. Moreover, an association between the presence of smokers and occurrence of wheezing episodes was found. The results highlight the importance of providing good air quality in infants’ bedrooms and avoiding parental in-house smoking habits. Although the exploratory nature of this study and the low participation rate limit the generalization to the region and to the rest of the country, it provides useful clues that can help inform future studies and public health policies for this pollution-affected region. Furthermore, our results are important to design future larger studies focused on the impact of IAQ on infants’ respiratory health.

## Figures and Tables

**Table 1:** Prevalence of wheezing by gender (*n* = 131).

		Boys	Girls	*p*-Value
**Mean age in months (SD)**		11.0 (8.8)	12.3 (10.6)	0.776
**Wheezing**	Never	49 (72.1%)	37 (58.7%)	0.108
	From birth	19 (27.9%)	26 (41.3%)	
**Wheezing in the past 12 months**		16 (23.5%)	23 (36.5%)	0.267

**Table 2:** Bedroom characteristics (*n* = 131) (adapted from: [30]).

	Total		Boys		Girls		
	*n*	%	*n*	%	*n*	%	*p*-Value
**Location**							
Rural	14	10.7	4	5.9	10	15.9	0.064
Urban	117	89.3	64	94.1	53	84.1	
**Smokers in house**							
Yes	40	30.5	20	29.4	20	31.7	0.772
No	91	69.5	48	70.6	43	68.3	
**Children’s bedrooms characteristics**							
**Floor location**							
Ground Floor	29	22.1	12	17.6	17	27.0	0.298
1st floor	38	29.0	23	33.8	15	23.8	
2nd floor or more	64	48.9	33	48.5	31	49.2	
**Visible mold**							
Yes	39	29.8	18	26.5	21	33.3	0.391
No	92	70.2	50	73.5	42	66.7	
**Proximity to pollution site**							
Yes	32	24.4	14	20.6	18	28.6	0.288
No	99	75.6	54	79.4	45	71.4	
**Decoration**							
Paint	119	90.8	62	91.2	57	90.5	0.898
Paint and paper	10	7.6	5	7.4	5	7.9	
Other	2	1.5	1	1.5	1	1.6	
Area m^2^ (SD)	13.4 (2.8)		13.0 (2.9)		13.9 (2.8)		0.088
Room density m^2^/person (SD)	5.9 (2.9)		5.8 (3.1)		6.15 (2.8)		0.683
**Used in the past 48 h**							
**Bedroom used for hygiene**							
Yes	74	56.5	39	57.4	35	55.6	0.836
No	57	43.5	29	42.6	28	44.4	
**Air conditioned**							
Yes	15	11.5	8	11.8	7	11.1	0.907
No	116	88.5	60	88.2	56	88.9	
**Oil radiator**							
Yes	19	14.5	10	14.7	9	14.3	0.946
No	112	85.5	58	85.3	54	85.7	
**Floor cleaning products**							
Yes	28	21.4	11	16.2	17	27.0	0.132
No	103	78.6	57	83.8	46	73.0	
**Furniture cleaning products**							
Yes	8	6.1	4	5.9	4	6.3	0.911
No	123	93.9	64	94.1	59	93.7	
**Recently washed clothes**							
Yes	41	31.3	24	35.3	17	27.0	0.305
No	90	68.7	44	64.7	46	73.0	
**Air cleaner/dehumidifier**							
Yes	13	9.9	6	8.8	7	11.1	0.662
No	118	90.1	62	91.2	56	88.9	
**Use of anti-insect/anti-moth products**							
Yes	6	4.6	1	1.5	5	7.9	0.077
No	125	95.4	67	98.5	58	92.1	
**Window opening frequency**							
No more than once a month	4	3.1	1	1.5	3	4.8	0.155
No more than once a week	4	3.1	1	1.5	3	4.8	
2 or more per week	33	25.2	19	27.9	14	22.2	
Once a day	69	52.7	40	58.8	29	46.0	
2 or more per day	21	16.0	7	10.3	14	22.2	

**Table 3:** Indoor air quality parameters, outdoor air quality parameters, and indoor/outdoor (I/O) ratios. (Legend: *n* = 131, except for § where *n* = 128 valid cases; ⸸ where *n* = 109 valid cases; ¥ where *n* = 121 valid cases).

		Temperature, °C	Relative Humidity, %	CO_2_, ppm	TVOC, mg/m^3^	CO, mg/m^3^
Reference Values: [21,22,23,24]		20–24	30–70	<1800	<0.3	<5.8
% rooms outside references		77.9	88.5	19.8	48.1	0
Indoor	Mean (SD)	18.4 (2.1)	75.9 (6.8)	1260.8 (1046.7)	3.1 (4.9)	0.2 (0.6)
	Median	18.3	75.5	951.0	0.2	0.0
	Range (min–max)	13.9–25.4	56.8–95.9	1.0–5545.0	0–23.1	0.0–3.5
	P25–P75	16.9–19.8	71.8–80.8	597.0–1595.0	0.0–5.0	0.0–0.0
Outdoor	Mean (SD)	18.5 (3.5)	70.1 (10.9)	614.9 (672.3)	2.0 (3.7)	0.1 (0.4)
	Median	18.1	70.2	449.0	0.0	0.0
	Range (min–max)	12.9–31.0	27.0–90.9	0–3229	0.0–13.3	0.0–2.3
	P25–P75	16.3–20.0	65.4–77.5	276.0–542.0	0.0–1.4	0.0–0.0
I/O Ratio	Mean (SD)	1.0 (0.2)	1.1 (0.2)	5.3 (12.9) ^§^	1.9 (2.8) ^⸸^	1.0 (0.2) ^¥^
	Median	1.0	1.1	2.2 ^§^	1.0 ^⸸^	1.0 ^¥^
	Range (min–max)	0.5–1.4	0.8–2.7	0.2–127.5 ^§^	0.0–19.2 ^⸸^	0.0–2.0 ^¥^
	P25–P75	0.9–1.1	1.0–1.1	1.5 ^§^–3.3 ^§^	1.0 ^⸸^–1.3 ^⸸^	1.0 ^¥^–1.0 ^¥^

TVOC, total volatile organic compound; I/O, indoor/outdoor ratio.

**Table 4:** Episodes of wheezing by sex and exposure to TVOC (*n* = 131).

	Episodes of Wheezing	Exposure to TVOC ≤ 0.3 mg/m^3^ *n* (%)	Exposure to TVOC > 0.3 mg/m^3^ *n* (%)
Boys	Never	25 (67.6%)	24 (77.4%)
	Between 1 and 3 episodes	5 (13.5%)	2 (6.5%)
	More than 3 episodes	7 (18.9%)	5 (16.1%)
Girls	Never	20 (64.5%)	17 (53.1%)
	Between 1 and 3 episodes	5 (16.1%)	8 (25.0%)
	More than 3 episodes	6 (19.4%)	7 (21.9%)

**Table 5:** Odds ratio and adjusted odds ratio for association of exposure to indoor smoking and different number of episodes of wheezing.

	Presence of Indoor Smokers		TVOC above 0.3 mg/m^3^		
	OR	aOR	OR	aOR	^⸸^ aOR
Never	1	1	1	1	1
Between 1 and 3 episodes	3.66	4.202	1.098	1.264	0.770
	[1.335–10.000]	[1.122–15.625]	[0.415–2.907]	[0.349–4.587]	[0.196–3.021]
	(*p* = 0.012)	(*p* = 0.033)	(*p* = 0.851)	(*p* = 0.721)	(*p* = 0.708)
More than 3 episodes	n.a	n.a	1.013 [0.415–2.469] (*p* = 0.977)	0.927 [0.337–2.545] (*p* = 0.883)	1.014 [0.355–2.899] (*p* = 0.979)

OR, odds ratio; aOR, adjusted odds ratio: area (m^2^); age (months); sex; temperature °C; relative humidity %; ^⸸^ aOR, adjusted odds ratio: presence of indoor smokers; area (m^2^); age (months); sex; temperature °C; relative humidity %; n.a. not available due to the low number of cases.
